# Effect of C-Terminal Residues of Aβ on Copper Binding Affinity, Structural Conversion and Aggregation

**DOI:** 10.1371/journal.pone.0090385

**Published:** 2014-03-03

**Authors:** Shu-Hsiang Huang, Shyue-Chu Ke, Ta-Hsin Lin, Hsin-Bin Huang, Yi-Cheng Chen

**Affiliations:** 1 Department of Medicine, MacKay Medical College, New Taipei City, Taiwan; 2 Department of Physics, National Dong Hwa University, Hualien, Taiwan; 3 Department of Medical Research & Education, Taipei Veterans General Hospital, Taipei city, Taiwan; 4 Institute of Biochemistry, National Yang-Ming University, Taipei, Taiwan; 5 Institute of Molecular Biology, National Chung-Cheng University, Chiayi, Taiwan; Russian Academy of Sciences, Institute for Biological Instrumentation, Russian Federation

## Abstract

Many properties of Aβ such as toxicity, aggregation and ROS formation are modulated by Cu^2+^. Previously, the coordination configuration and interaction of Cu^2+^ with the Aβ N-terminus has been extensively studied. However, the effect of Aβ C-terminal residues on related properties is still unclear. In the present study, several C-terminus-truncated Aβ peptides, including Aβ1-40, Aβ1-35, Aβ1-29, Aβ1-24 and Aβ1-16, were synthesized to characterize the effect of Aβ C-terminal residues on Cu^2+^ binding affinity, structure, aggregation ability and ROS formation. Results show that the Aβ C-terminal residues have effect on Cu^2+^ binding affinity, aggregation ability and inhibitory ability of ROS formation. Compared to the key residues responsible for Aβ aggregation and structure in the absence of Cu^2+^, it is more likely that residues 36–40, rather than residues 17–21 and 30–35, play a key role on the related properties of Aβ in the presence of Cu^2+^.

## Introduction

Alzheimer's disease (AD) is a neurodegenerative disorder that destroys neuronal cells in the human brain [1], [2]. Numerous reports have shown that one of the pathological hallmarks in the brain of AD patients is the cerebral senile plaques [1], [2]. Senile plaques contain 90% of β-amyloid peptide (Aβ), including Aβ1-40 and Aβ1-42, which is a proteolytic product of amyloid precursor protein (APP) [3]–[5]. The others remained in senile plaques include apolipoproteins E, lipids from membranes of degenerated portions of neuron, and abnormally high concentration of metal ions such as Cu^2+^, Zn^2+^, or Fe^2+^ [6], [7].

In the amyloid cascade hypothesis, the Aβ aggregates are proposed to be the main toxic species and the cause of AD [8], [9]. Aβ adopts a β-sheet conformation in the aggregated state, and the amyloid aggregates can induce free radical formation and subsequently cause neuronal death [8], [9]. Among the Aβ aggregates, oligomer and protofibril rather than mature amyloid fibril have been demonstrated to be the most toxic species to neurons [10]–[12]. The aggregation and toxicity of Aβ is well correlated with its sequence and structure [13]–[15]. Previous studies using different Aβ fragments or truncated Aβ peptides reported that residues 17–21 and 30–35 are the most important regions for aggregation and neurotoxicity [14], [15].

The deposition of Aβ has been shown to be modulated by metal ions [6], [7], particularly Cu^2+^. Abnormally high concentrations of Cu^2+^ have been found in cerebral amyloid-deposits of AD patients [6]. It has been shown that Cu^2+^ is bound to Aβ [6], [16]. Either one or two Cu^2+^ bound to Aβ peptide has been proposed. The binding site is mainly located at the N-terminus of Aβ, particularly the three histidine residues (His6, His13 and His14), and forms a 3N1O coordination configuration [16], [17]. The reported Cu^2+^ binding affinities for monomeric Aβ vary widely between micromolar and nanomolar [18]. The effect of Cu^2+^ ion on Aβ has been shown to be twofold, the first is to accelerate the aggregation of Aβ, [19], [20], and the second is to induce the formation of reactive oxygen species (ROS) [18], [21].

The role of Aβ coordinated with Cu^2+^ on the free radical has still been under debate. Both pro-oxidant and antioxidant roles for Aβ associated with the ROS produced by Cu^2+^ have been suggested [21]–[23]. Although early studies suggested that Aβ peptides can spontaneously produce free radicals [14], [18], [19], [22], several studies have shown that Aβ required the presence of Cu^2+^ to produce ROS [18], [21], [22]. The possible mechanism of ROS formation may be through a series of electron transfer reactions when Cu^2+^ binds to Aβ [18], [21]. The ROS induced by Aβ/Cu^2+^ aggregates is reduced by addition of other antioxidants or Cu-selective chelators [17], [23]–[25]. As opposed to the pro-oxidant role, other studies have proposed an antioxidant activity of Aβ [26]–[28]. In particular, monomeric Aβ1-40 has been shown to inhibit neuronal death caused by Cu^2+^ induced oxidative damage [27], [28]. Furthermore, Viles group has demonstrated that Aβ does not silence the redox reaction of Cu^2+^ via chelation but react with the hydroxyl radicals produced by Cu2^+^/ascorbate and quench the harmful oxidative species [26].

The effect of Aβ sequence on structure, aggregation ability and ROS formation in the absence of Cu^2+^ has been extensively studied [14], [15]. In general, residues 17–21 and 30–35 are identified as the key region responsible for aggregation and neurotoxicity [14], [15]. In the presence of Cu^2+^, the interaction and coordination configuration of Aβ/Cu^2+^ complex have been characterized [16]–[18]. However, most of these studies have focus on the elucidation of interaction and coordination configuration of the Aβ N-terminus with Cu^2+^. So far, the effect of Aβ C-terminal residues on Cu^2+^ binding affinity, aggregation ability and ROS formation in the presence of Cu^2+^ has yet been studied.

In the present study, we investigated the effect of Aβ C-terminal residues on Cu^2+^ binding affinity, structure, aggregation ability and ROS formation. Full length Aβ1-40 and several C-terminus-truncated Aβ peptides, including Aβ1-35, Aβ1-29, Aβ1-24 and Aβ1-16 were synthesized and used to characterize these subjects. Our results indicated that, though the major Cu^2+^ binding site is located at N-terminus of Aβ, the C-terminal residues of Aβ, particularly residues 36–40, have a significant effect on the binding affinity of Cu^2+^, conformation, aggregation ability and the inhibitory ability of ROS driven by Cu^2+^.

## Materials and Methods

### Synthesis and purification of Aβ peptides

The synthesis of Aβ peptides, including Aβ1-40, Aβ1-35, Aβ1-29, Aβ1-24 and Aβ1-16, were performed in a solid-phase peptide synthesizer (PS3, Protein Technologies, Inc., AZ) using the FMOC protocol with HMP resin. After cleavage from the resin with a mixture of trifluoroacetic acid/H_2_O/ethanedithiol/thioanisole/phenol, the peptides were extracted with 1∶1 (v:v) ether: H_2_O containing 0.1% 2-mercaptothanol. The synthesized Aβ peptides were purified using a C18 reverse-phase column with a linear gradient from 0% to 78%. Peptide purity was over 95% as identified by MALDI–TOF mass spectrometer. One mg of purified Aβ peptides was dissolved in 1 ml trifluoroethanol, and centrifuged (20,000×*g*) to sediment the insoluble particles. This Aβ solution was then dried under N_2_ gas and resuspended in 1 ml phosphate buffer, pH 7.4, to provide a stock solution, and stored at −80°C until used.

### Copper binding affinity assay

Tyrosine fluorescence spectroscopy was used to characterize the binding affinity of Cu to Aβ [28]. Before measurements, the stock solution containing the different C-terminal truncated Aβ peptides was diluted in Dulbecco's PBS, pH 7.0 to a final peptide concentration of 10 μM with different molar ratios of CuCl_2_. Spectra were collected on a microplate reader (FlexStation 3, MD). The excitation and emission wavelength was 278 and 305 nm, respectively. The intensity change at 305 nm was used to calculate the binding constant. Previously, the number of Cu ion bound to Aβ has been debated. Either one or two Cu ion has been proposed to bind to Aβ [29], and there is no two-Cu/Aβ complex structure available. Two-degenerate scheme for either one- or two-Cu binding modes was hence considered and applied to calculate the binding constant.

For the one-Cu^2+^ binding mode, the general equation for Cu^2+^ binding is as follows: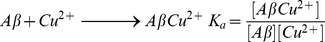, the degree of saturation, Y, can be written as



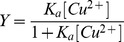





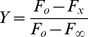
, where I_o_ and I_x_ are the fluorescence intensity in Cu-free and Cu-bound state, respectively. I_∞_ is the fluorescence intensity at saturation state, [Cu^2+^] is the copper concentration, n is the copper binding number and *K_a_* is the association constant.

For the two-Cu^2+^ binding mode, the two Cu^2+^ ions are bound to Aβ located at the N-terminal His-pocket. The general equation for Cu^2+^ binding is as follows:
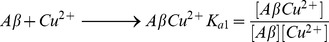





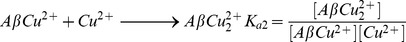



The tyrosine fluorescence spectrum at any concentration is the net combination of the Cu-free and Cu-bound forms weighted by their concentrations. Two general models based on linked two-site binding are proposed.

The first model (dependent mode) is one in which the two Cu binding sites interact so that the second Cu^2+^ ion binds with a different binding constant than the first. The degree of saturation for the dependent mode can be described as follows [30]:
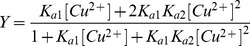



The second model (independent mode) assumes that the two binding sites, due to the different structure or accessibility, are independent with each other and should have equal binding constant (K_a1_ =  K_a2_) for the two Cu^2+^ ions [30]. The degree of saturation for the independent mode is described as follows:
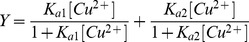



The related parameter was calculated using the nonlinear curve fitting function in the Origin6.0 program (Microcal Software, Inc., Nothampton, MA). This nonlinear fitting program uses the Levenberg-Marquardt nonlinear least-squares fitting algorithm. In the initial fitting stage, the Simplex method, which was set to 100 cycle runs, was used to calculate the initial parameter for further nonlinear curve fitting. A 0.95 confidence level was set to constrain the quality of curve fitting. The final fitting parameters were obtained when the value of χ^2^ was less than 0.05 and the parameters and errors for the parameters reached the convergent and steady state.

### Circular dichroism (CD) spectroscopy

Thirty μM of fresh peptide samples, diluted from the stock solution in phosphate buffer, pH 7.0, in the presence or absence of 30 μM Cu^2+^ were used for CD measurements. CD spectra were recorded, within 1 hr after samples prepared, using either an Aviv 420 spectropolarimeter or synchrotron radiation CD (04B1) in the national synchrotron radiation center, Taiwan. All measurements were performed in a quartz cell with pathlength of 0.1 cm. Spectra were collected at the wavelengths from 190 to 260 nm in 0.5 nm increments. Reported CD spectra were the average from three repeats of samples. The reported CD spectra were corrected for baseline using the solution of PBS buffer, pH 7.0 and Cu^2+^ ions. The secondary structure analysis was calculated using CDSSTR program in Dicroweb website [31].

The β-sheet propensity is defined as 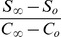. *S_o_* and *S_∞_* represent the percentage of β-sheet content in Cu-free and saturated Cu-bound state, respectively. *C_o_* and *C_∞_* are the concentrations of Cu^2+^ in Cu-free and saturated Cu-bound state, respectively.

### Aggregation assay

The aggregation process of Aβ peptides in the presence or absence of Cu^2+^ was assessed by the turbidity assay. Thirty μM of Aβ peptides were placed in a 96-well plate and incubated in the presence or absence of 30 μM CuCl_2_ at 37°C. Turbidity was measured using a microplate reader (FlexStation 3, MD) at a wavelength of 450 nm.

### ROS assay

ROS (H_2_O*_2_*) level induced by Aβ/Cu^2+^ was analyzed using the dichlorofluoresein diacetate (DCFH-DA) assay [17]. Dichlorofluorescein diacetate was dissolved in 100% dimethyl sulfoxide (DMSO), deacetylated with 1∶1 (v/v) 4 M NaOH for 30 min, and then neutralized (pH 7.2) to a final concentration of 200 μM as stock solution. This stock solution was kept on ice and in the dark until use. The reaction was carried out in a 96-well plate (100 μl/well) in Dulbecco's PBS, pH 7.2, containing the designed concentrations of Aβ peptides, 30 μM of CuCl_2_, 20 µM deacylated DCF and 5 μM horseradish peroxidase, and incubated at 37°C for 1 hr. Measurements were performed on the day of sample prepared. Fluorescence readings were recorded on the microplate reader (Flexstation3, MD). The excitation and emission wavelengths were 485 and 530 nm, respectively.

### Electron paramagnetic resonance (EPR) spectroscopy

Samples containing 300 μM of Aβ peptides and Cu^2+^ ions in 30% glycerol phosphate buffer, pH 7.2, freshly prepared from peptide stock solution were employed for EPR spectroscopic measurements. EPR spectra were obtained at X-band using a Bruker EMX ER073 spectrometer equipped with a Bruker TE102 cavity and an advanced research system continuous-flow cryostat (4.2–300 K). During EPR experiments, the sample temperature was maintained at 10 K. The microwave frequency was measured with a Hewlett-Packard 5246L electronic counter.

### Transmission Electron Microscopy (TEM)

A transmission electron microscopy (JEM-2000 EXII, JEOL, Japan) with an accelerating voltage of 100 KeV was used to analyze the morphology of Aβ peptides incubated with Cu^2+^. Ten microliters of sample with the different Aβ peptides and Cu^2+^ ions in 1∶1 molar ratio used for the aggregation assay was used. Each peptide sample was placed onto a carbon-coated 200 mesh copper grid (Pelco, Ca, USA). Excess solution was wicked dry with tissue paper, and the sample was negatively stained with 5 ml of 2% uranyl acetate for 30 seconds. After TEM analyses, these copper grids coated with Aβ samples used for TEM analyses were further treated with 50 μL 1 mM EDTA solution three times to strip off Cu^2+^ ions and then incubated at 37°C for 24 hrs. These copper grids coated with Aβ samples treated with EDTA were then conducted for TEM analyses to observe the morphology of Aβ peptides in absence of Cu^2+^.

## Results

### Correlation of Cu binding affinity and Aβ sequence

The aggregation and toxicity of Aβ has been demonstrated to be modulated by Cu^2+^ [6], [7], [17], [18], [21]. The interaction of Cu^2+^ with Aβ N-terminus has been extensively studied [17], [18], [32]–[34]. The number of Cu^2+^ bound to Aβ has been debated which either one or two Cu^2+^ has been proposed [17], [18], [29]. On the other hand, the effect of C-terminal residues on Cu^2+^ binding affinity and other properties has yet to be studied. In order to unveil the effect of C-terminal residues on Cu^2+^ binding affinity and other properties, several Aβ peptides, including Aβ1-40, Aβ1­35, Aβ1­29, Aβ1­24, Aβ1­16 and Aβ25-35, were synthesized and used to characterize the correlation with Cu^2+^ binding affinity, structural changes and aggregation ability.

**Figure 1:**
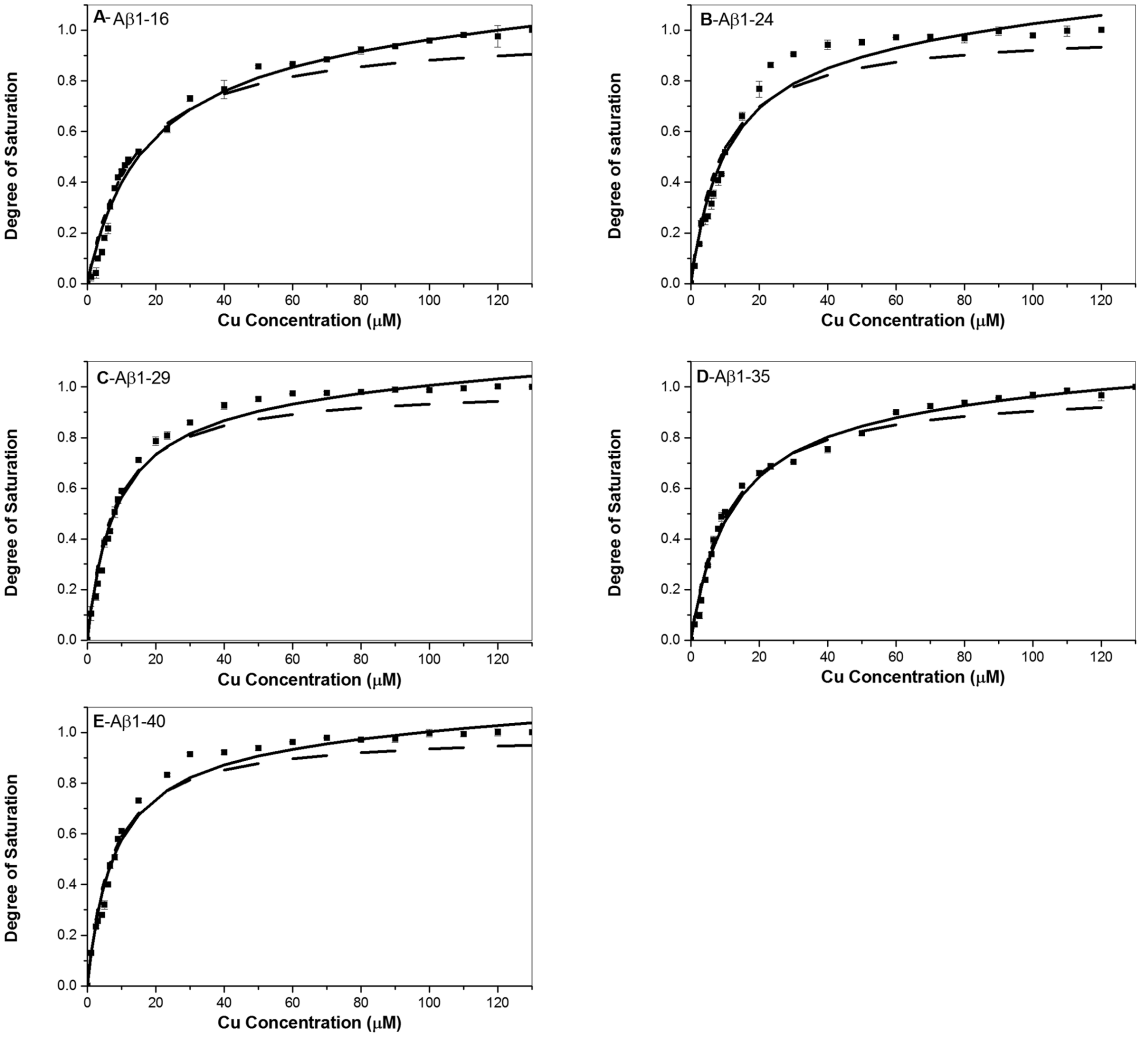
Tyrosine fluorescence spectra for the determination of Cu^2+^ binding affinity. (A) Aβ1-16, (B)Aβ1-24, (C)Aβ1-29, (D)Aβ1-35, (E)Aβ1-40. The concentration of Aβ peptides was 10 µM. The solid lines represent the best fitting curve using the independent two-Cu mode, whereas dot lines show the fitting curve simulated using one-Cu mode as depicted in the section of material and methods.

To characterize the Cu^2+^ binding affinity, tyrosine fluorescence spectroscopy was used to determine the Cu^2+^ binding constants. Figures 1 (A–E) show the tyrosine fluorescence titration curves as a function of tyrosine fluorescence intensity vs. Cu^2+^ concentration for Aβ1-40, Aβ1­35, Aβ1­29, Aβ1­24, and Aβ1­16, respectively. Both one-Cu and two-Cu binding modes were applied to estimate the Cu^2+^ binding constants. As shown in Fig. 1, the two-Cu mode (solid line) shows to fit the titration curve better than the one-Cu mode (dot line) for all Aβ peptides, indicating that the Cu^2+^ binding site is more likely to locate two ions instead of one ion for all Aβ peptides. For the two-Cu mode, we further tested if the two Cu^2+^ ions bound to Aβ are dependent or independent of each other. As shown in Figs. 1 (A–E), for all Aβ peptides, the non-linear fitting curves were only convergent by using the dependent mode, suggesting that the binding constant of two Cu^2+^ ions should be different for each other.

The calculated binding constants are summarized in Table 1. The *K_a1_* value was approximately hundredfold higher than the *K_a2_* value for all Aβ peptides, indicating that the first Cu binds to Aβ much stronger that the second Cu does. The *K_a1_* value was in the range of 0.06–0.13 μM, and the *K_a2_* value was in the range of 0.0007–0.0013 μM. In general, both *K_a1_* and *K_a2_* were dependent on sequence. The value of *K_a1_* was increased with an increase of Aβ C-terminal residues, except of Aβ1-35. The *K_a1_* value of Aβ1-40 was approximately twofold higher than that of Aβ1-16. In contrast, the trend of *K_a2_* value was opposite to that of *K_a1_* value which the *K_a2_* values of Aβ1-24 and Aβ1-16 were higher than those of Aβ1-29, Aβ1-35 and Aβ1-40. The *K_a2_* value of Aβ1-24 was approximately twofold higher than that of Aβ1-35. The *K_a1_*value for Aβ peptides was in the order of Aβ1-40≥ Aβ1-29≥ Aβ1-35≈ Aβ1-24> Aβ1-16, whereas the *K_a2_* value for Aβ peptides was in the order of Aβ1-24≥ Aβ1-16≥ Aβ1-29≈ Aβ1-40≈ Aβ1-35.

**Table 1:** The estimated copper (II) binding constant using one-Cu and dependent two-Cu models for the different Aβ peptides.

	*One-Cu*	*Two-Cu (dependent)*			
	*K_a_*	*K_a1_*	*K_a2_*	*R^2^*	*χ*
Aβ1-16	0.07±0.02	0.06±0.01	0.0011±0.0001	0.98	0.0019
Aβ1-24	0.10±0.04	0.09±0.02	0.0013±0.0004	0.97	0.0038
Aβ1-29	0.12±0.03	0.11±0.02	0.0009±0.0003	0.98	0.0016
Aβ1-35	0.10±0.01	0.09±0.01	0.0007±0.0001	0.99	0.0010
Aβ1-40	0.14±0.02	0.13±0.01	0.0008±0.0002	0.98	0.0019

### EPR spectra of Aβ/Cu^2+^ complexes

As we showed that the C-terminal residues of Aβ can affect the Cu^2+^ binding affinity, it is of interest to examine if the interaction of the C-terminal residues with Cu^2+^ alters the coordination configuration of Aβ/Cu^2+^. In order to characterize the coordination configuration, EPR spectroscopy was used to determine the coordination configuration of Aβ/Cu^2+^ for the different C-terminus-truncated Aβ peptides.

**Figure 2:**
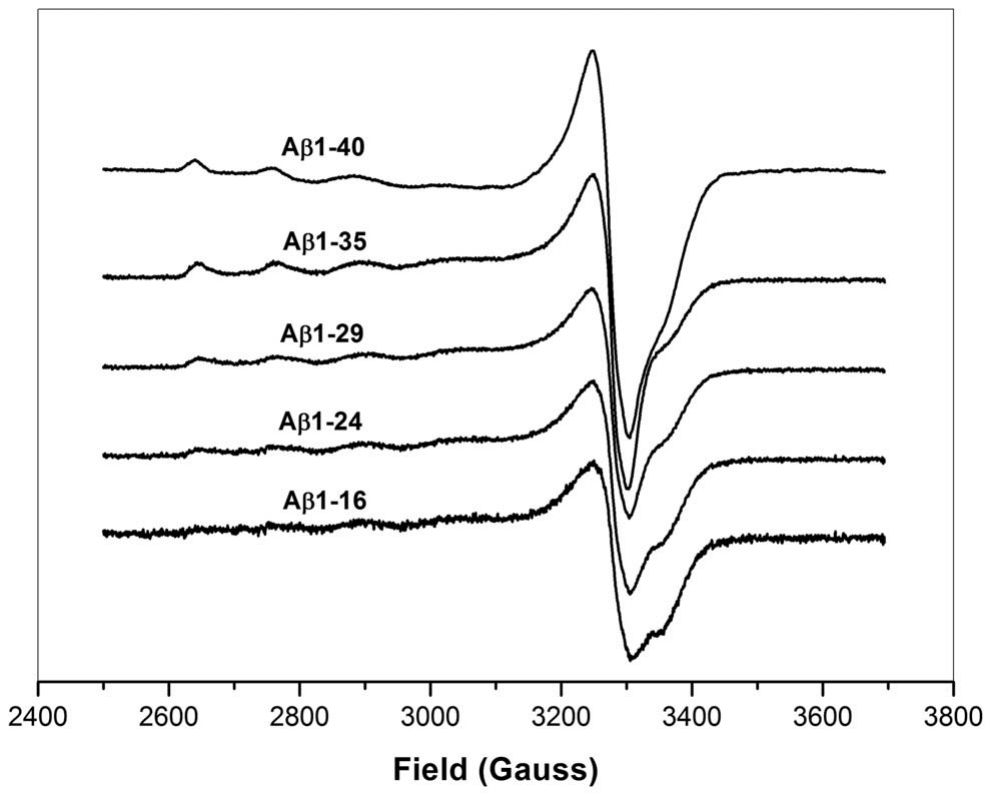
EPR spectra for the characterization of coordination configuration. EPR spectra for Aβ1-16 (black), Aβ1-29 (red), Aβ1-35 9greeen), and Aβ1-40 (blue) in the presence of Cu^2+^. The characteristic g_||_ and g_⊥_ hyperfine peaks appeared spectra represent that Cu^2+^ ion coordinates with Aβ peptides in a similar coordination configuration.

Figure 2 shows the EPR spectra for Cu^2+^ with Aβ1-40, Aβ1-35, Aβ1-29, Aβ1-24 and Aβ1-16. The EPR parameters of g_⊥_, g_||_ and A are listed in Table 2. It can be seen that the hyperfine peaks of EPR spectra for the different Aβ peptides showed a similar pattern. The estimated g_⊥_, g_||_ and A parameters for the different Aβ peptides were similar and very close to literature report in aqueous condition except of Aβ1-16 [33], [34]. The values of g_⊥_, g_||_ and A were approximately 2.060, 2.266 and 169 for all Aβ peptides except of Aβ1-16. The value of A parameter for Aβ1-16 is slightly lower than that for the other Aβ peptides, indicating that the Cu^2+^ binding affinity of Aβ1-16 is relatively weak compared to the other Aβ peptides. In general, our results indicate that the coordination configuration of Cu^2+^ for Aβ peptides adopt mainly a 3N1O ligand-donor-atom set [17], [18], [29], [32], [33], and the main coordination configuration of Aβ/Cu^2+^, located at the N-terminus was not significantly altered by the association of C-terminus of Aβ.

**Table 2:** The estimated EPR parameters of Aβ/copper (II) complex for the different Aβ peptides.

	g_⊥_	g_||_	*A*
Aβ1-16	2.061	2.266	160
Aβ1-24	2.060	2.266	168
Aβ1-29	2.060	2.266	169
Aβ1-35	2.060	2.265	168
Aβ1-40	2.060	2.265	170

### Secondary structure of Aβ peptides in the presence of Cu^2+^

Previous results show that the increase of C-terminal residues increased the Cu^2+^ binding affinity but did not cause any significant change of Aβ-Cu^2+^ coordination configuration. However, several studies have shown that the binding of Cu^2+^ to Aβ can induce a conformational conversion from either helix or random coil into β–sheet [19], [20]. Therefore, the effect of C-terminal residues on the secondary structure of Aβ peptides in the presence of Cu^2+^ was examined by using CD spectroscopy.

**Figure 3:**
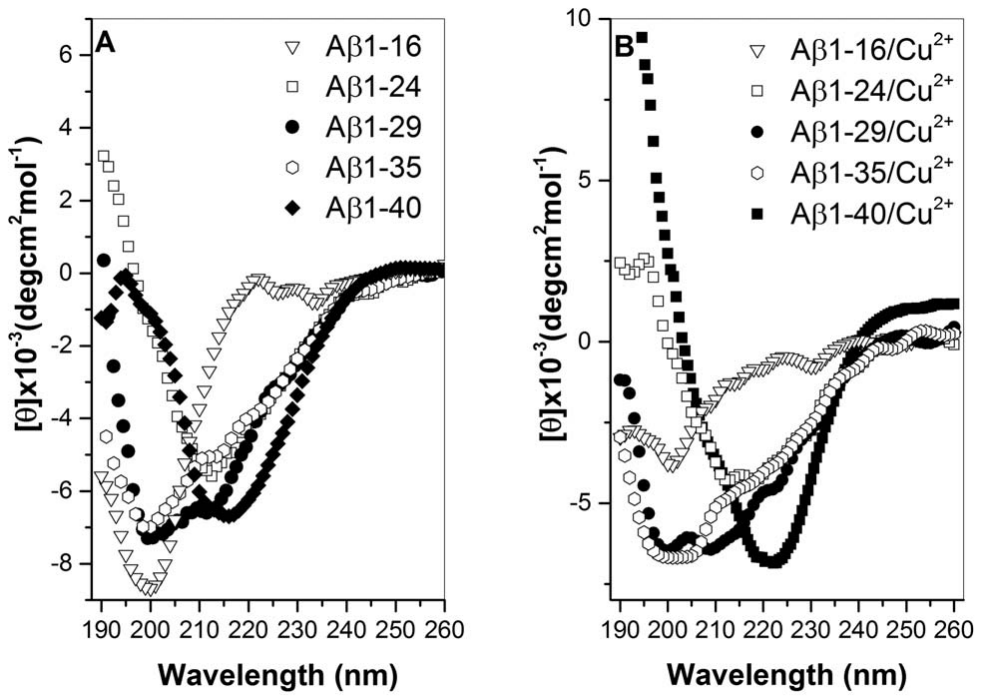
Circular dichroism spectra of Aβ peptides. CD spectra for different Aβ peptides, (▿) Aβ1-16, (□) Aβ1-24, (•) Aβ1-29, (○) Aβ1-35, (▪) Aβ1-40, in the absence (A) and presence (B) of Cu^2+^. The concentration for both Aβ peptides and Cu^2+^ used in measurements was 30 µM. A normalized root mean square standard deviation (NRMSD) parameter was introduced to indicate for the quality between observed and calculated CD spectra.

Figures 3 (A) and (B) show the CD spectra for the different Aβ peptides in the absence or presence of Cu^2+^ ions, respectively. Table 3 summarizes the estimated content of secondary structure. In general, in the absence of Cu^2+^, all Aβ peptides adopt a high percentage of random coil. Aβ1-16 contained the highest percentage of random coil (72%) and the lowest percentage of β-sheet (24%), whereas other Aβ peptides contained a similar secondary structure content, 30–34% of β-sheet, 61–64% of random coil and 4–5% of α-helix. In the presence of Cu^2+^, the secondary structure content for Aβ1-35, Aβ1-29, and Aβ1-24 peptides was similar to that obtained in the absence of Cu^2+^. For Aβ1-16, the β-sheet percentage was slightly increased (27%), and the random coil percentage was slightly decreased (70%). In contrast to other C-terminus-truncated Aβ peptides, the secondary structure of Aβ1-40 showed a dramatic change while adding the Cu^2+^ ions. The β-sheet content of Aβ1-40 increased from 34% to 47%, and the random coil percentage of Aβ1-40 decreased from 61% to 50% in the presence of Cu^2+^.

**Table 3:** The content of secondary structure for the different Aβ peptides in the presence or absence of Cu^2+^ as calculated from CD spectra.

	Α-helix (%)	B-sheet (%)	Random coil (%)	NRMSD*
Aβ1-16	4	24	72	0.01
Aβ1-24	5	31	64	0.06
Aβ1-29	5	30	65	0.10
Aβ1-35	5	30	65	0.12
Aβ1-40	5	34	61	0.03
Aβ1-16/Cu^2+^	3	27	70	0.11
Aβ1-24/Cu^2+^	3	34	63	0.008
Aβ1-29/Cu^2+^	3	35	62	0.14
Aβ1-35/Cu^2+^	4	34	62	0.11
Aβ1-40/Cu^2+^	3	47	50	0.02

*NRMSD (normalized root mean square standard deviation)  =  [(θ_obs_(λ)-θ_cal_(λ))^2^/(θ_obs_(λ))^2^]^1/2^.

**Figure 4:**
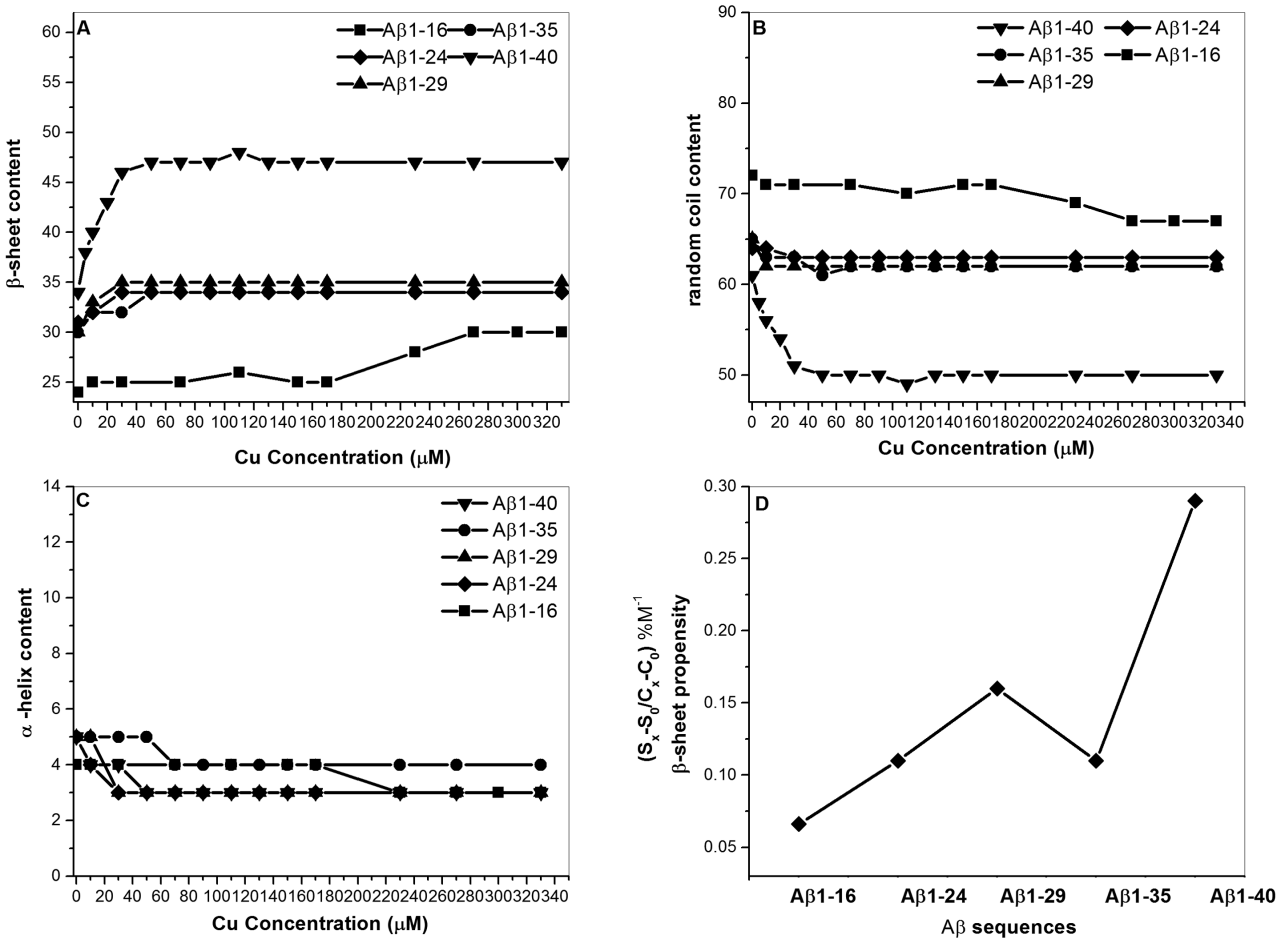
The plot of secondary structure content vs. Cu^2+^ concentration. The plot for different Aβ peptides, (▪) Aβ1-16, (♦) Aβ1-24, (▴) Aβ1-29, (○) Aβ1-35, (▾) Aβ1-40 and (A) β–sheet percentage, (B) random coil percentage, and (C) α-helx. (D) The plot of β–sheet propensity vs. Aβ peptides.

We further analyzed the correlation between secondary structure and Cu^2+^ concentration. The plot of secondary structural content (β-sheet, random coil and α-helix) vs. Cu^2+^ concentration for Aβ peptides is depicted in Figures 4 (A–C), respectively. In general, the contents of β-sheet and random coil for Aβ1-40, Aβ1-35, Aβ1-29, and Aβ1-24 were dependent on Cu^2+^ concentration, whereas the secondary structure content of Aβ1-16 was independent of Cu^2+^ concentration. For Aβ1-40, Aβ1-35, Aβ1-29, and Aβ1-24, the content of β-sheet structure increased with an increase of Cu^2+^ concentration (fig. 4 (A)), whereas the content of random coil decreased with an increase of Cu^2+^ concentration (fig. 4 (B)). The α-helix content showed no obvious change with the increase of Cu^2+^ concentration for all Aβ peptides (fig. 4 (C)). The change of β-sheet content for Aβ1-40 was more significant than those for other Aβ peptides. Furthermore, the relationship between β-sheet propensity and Aβ sequence in the presence of Cu^2+^ was also correlated by plotting the β-sheet propensity in the presence of Cu^2+^ vs. Aβ sequence. As it can be seen that has the β-sheet propensity of Aβ1-40 is significantly higher than those for other Aβ peptides. This is generally agreement with the result obtained from K_a1_ binding constant which Aβ1-40 has the higher Cu-binding affinity.

### Aggregation ability for Aβ peptides

It has been shown that the binding of Cu^2+^ can modulate the aggregation mechanism of Aβ [6], [14], [33]. As demonstrated by the present study, the conformational conversion into β-strand structure was dependent on Aβ C-terminal residues in the presence of Cu^2+^. In order to further characterize the effect of C-terminal residues on Aβ aggregative ability, we analyzed the aggregation profiles for the different C-terminus-truncated Aβ peptides in the presence of Cu^2+^.

**Figure 5:**
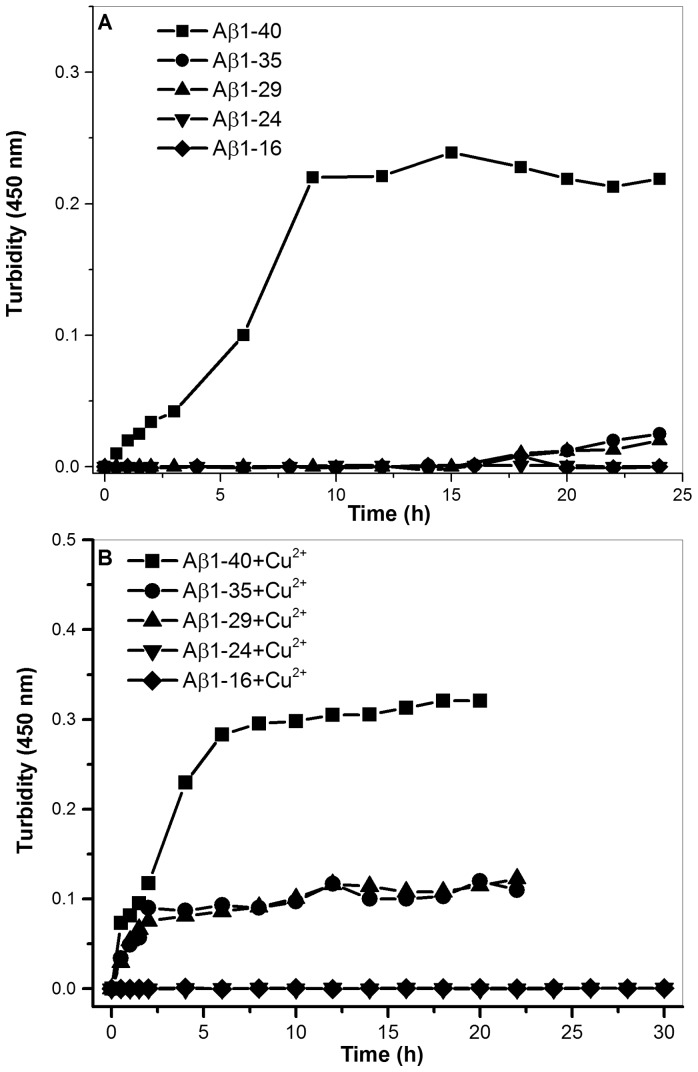
The aggregation profiles. The aggregation profile determined by turbidity assay in the absence (A) and the presence (B) of Cu^2+^ for different Aβ peptides, (⋄) Aβ1-16, (▾) Aβ1-24, (▴) Aβ1-29, (○) Aβ1-35, and (▪) Aβ1-40.

Figures 5 (A) and (B) show the aggregation profiles for Aβ1-40, Aβ1-35, Aβ1-29, Aβ1-24, and Aβ1-16 in the absence and presence of Cu^2+^, respectively. As shown in figure 5 (A), only Aβ1-40 was able to form aggregates in the absence of Cu^2+^, whereas the other Aβ peptides remained at nucleation state in the absence of Cu^2+^. In the presence of Cu^2+^, Aβ1-40, Aβ1-35 and Aβ1-29 were able to aggregate, whereas Aβ1-24 and Aβ1-16 remained at nucleation state (fig. 5 (B)), indicating that the C-terminal residues of Aβ, particularly residues 25–40, have effect on the aggregation in the presence of Cu^2+^. The aggregation rate for Aβ1-40 in the presence of Cu^2+^ was faster than the rates for Aβ1-35 and Aβ1-29. This result further suggests that the C-terminal residues, particularly 36–40, may play an important role on the aggregation mechanism of Aβ driven by Cu^2+^. In general, the aggregation ability is in the order of Aβ1-40> Aβ1-35≈ Aβ1-29>> Aβ1-24≈ Aβ1-16.

### TEM morphology of Aβ peptides

As shown in the previous sections that the C-terminal residues of Aβ have impact on Cu^2+^ binding affinity, secondary structure and aggregative ability. Therefore, we wondered if the C-terminal residues have any effect on the morphologies of fibrils formed by the Aβ peptides. To examine the effect of C-terminal residues on the morphologies of Aβ fibrils in the presence of Cu^2+^, transmission electronic microscopy was used to observe the fibril morphologies.

**Figure 6:**
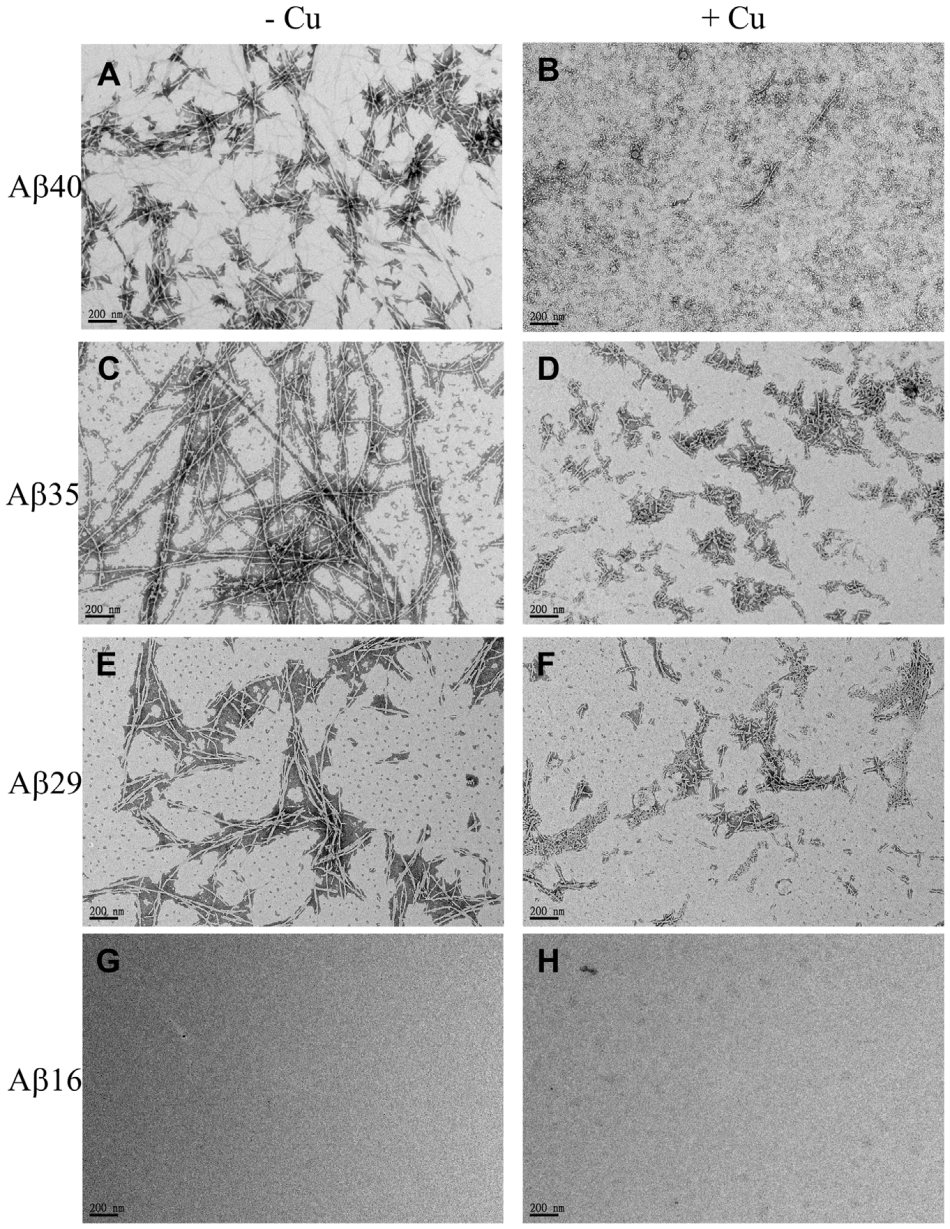
The TEM images of Aβ fibril morphologies. Images A, C, E and G represent the fibril morphologies for Aβ1-40, Aβ1-35, Aβ1-29 and Aβ1-16 with Cu^2+^ stripped off by EDTA, respectively. Images B, D, F and H represent the morphologies for Aβ1-40, Aβ1-35, Aβ1-29 and Aβ1-16 in the presence of Cu^2+^, respectively.

Figures 6 (A), (C), (E) and (G) show the morphologies for Aβ1-40, Aβ1-36, Aβ1-29 and Aβ1-16 in the presence of Cu^2+^ (molar ratio Aβ/Cu  = 1), respectively. It can be seen that most Aβ peptides formed a non-amyloid-like morphology in the presence of Cu^2+^, except of Aβ1-16 which did not form any amyloid fibrils. The same Aβ peptides/Cu^2+^ samples of the same spots were then treated with 1mM EDTA to strip off the Cu^2+^ ions and further incubated at 37°C and 24 hrs. After Cu^2+^ ions were depleted by EDTA, the morphology of Aβ peptides was then analyzed using TEM. Figures 6 (B), (D), (F) and (H) show the TEM images for morphologies of Aβ1-40, Aβ1-36, Aβ1-29 and Aβ1-16, respectively. The morphologies for Aβ1-40, Aβ1-35 and Aβ1-29 aggregates in the presence of Cu^2+^ are obviously very different from the morphologies of these peptides with Cu^2+^ stripped off by EDTA. In figure 6 (B), the fibril of Aβ1-36 was a typical amyloidogenic and network-like morphology after Cu^2+^ was stripped off by EDTA. On the other hand, the fibrils of both Aβ1-29 (fig. 6(E)) and Aβ1-40 (fig. 6(A)) with Cu^2+^ stripped off by EDTA were shorter and non-network-like morphology. For both Aβ1-16 and Aβ1-24, they did not form any fibril in the presence or absence of Cu^2+^.

### Correlation of H_2_O_2_ formation and Aβ sequence in the presence of Cu^2+^

The role of Aβ/Cu^2+^ on the formation of ROS is controversial. Both antioxidant and pro-oxidant roles for Aβ on the ROS formation in the presence of Cu^2+^ have been proposed [20]–[23]. In order to elucidate the effect of Aβ C-terminal residues on either antioxidant or pro-oxidant role, a DCF assay which usually detects the formation of H_2_O_2_ was used to measure the level of ROS for the different Aβ peptides in the presence of Cu^2+^.

**Figure 7:**
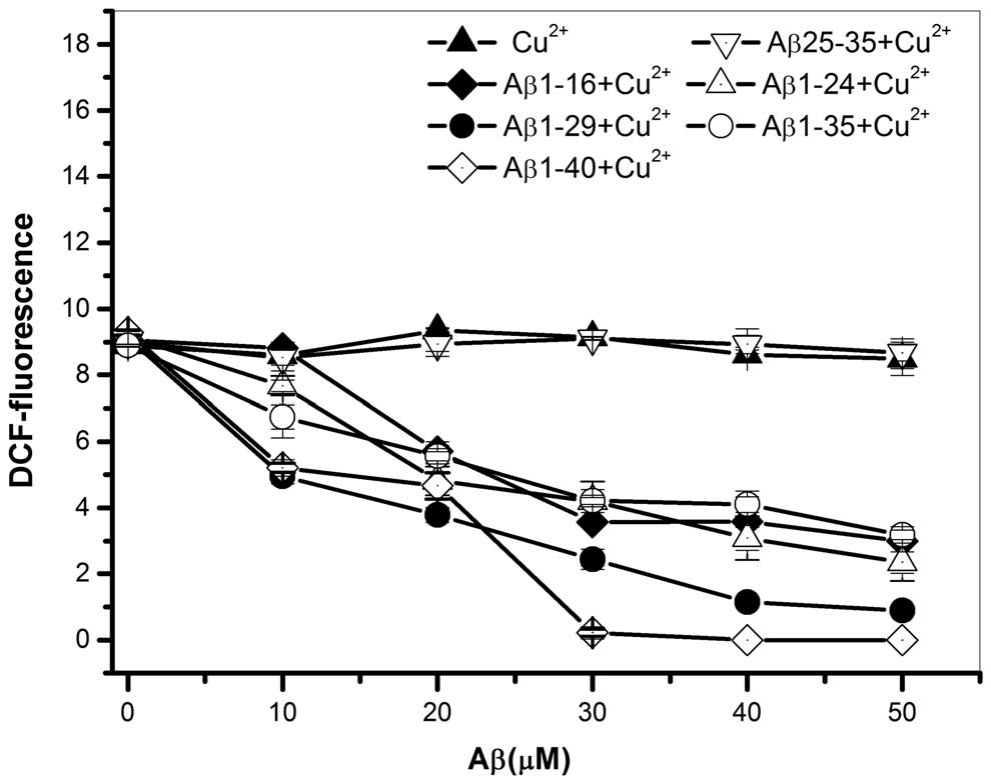
The plot of DCF fluorescence intensity vs. Aβ concentration. The plot for different Aβ peptides, (▴) 30 µM Cu^2+^ alone, (♦) Aβ1-16, (▵) Aβ1-24, (•) Aβ1-29, (○) Aβ1-35, (◊) Aβ1-40, and (▿) Aβ25-35 in the presence of 30 µM Cu^2+^. Instead of generating H_2_O_2_, most Aβ peptides, except of Aβ25-35, inhibit the generation of H_2_O_2_. All measurements were measured after the fresh prepared samples were incubated at 37°C for 1 hr.

Figure 7 shows the plot of DCF fluorescence intensity vs. Aβ concentration. For most C-terminus-truncated Aβ peptides, the DCF fluorescence intensity was decreased with an increase of Aβ concentration, indicating that the formation of H_2_O_2_ was inhibited by most Aβ peptides, except of Aβ25-35. For Aβ25-35, which lacks the Cu^2+^ binding site, did not show to inhibit the formation of H_2_O_2_. The H_2_O_2_ level was equal to that of Cu^2+^ only. For the comparison of inhibitory ability for these Aβ peptides, only full-length Aβ1-40 was able to completely inhibit the formation of H_2_O_2_ at the molar ratio of Aβ/Cu^2+^  = 1, whereas for other peptides such as Aβ1-35, Aβ1-29, Aβ1-24 and Aβ1-16, the formation of H_2_O_2_ was not completely inhibited at the molar ratio of Aβ/Cu^2+^  = 1. A higher peptide concentration was needed to completely reduce the H_2_O_2_ level to zero for C-terminus-truncated Aβ peptides.

Since the Cu^2+^ binding affinity was showed to be proportional with the length of C-terminal residues, taken together, our results further suggest that the Cu^2+^ binding affinity may be the key factor for the inhibition of H_2_O_2_ formation driven by Cu^2+^. In general, the inhibitory ability of ROS for these Aβ peptides was proportional with the binding affinity of Cu^2+^ and in the order of Aβ1-40> Aβ1-29> Aβ1-35≈ Aβ1-24≈ Aβ1-16>> Aβ25-35.

## Discussion

Amyloid cascade hypothesis proposes that the aggregated Aβ species are toxic to neurons and the main cause of Alzheimer's disease [6]. The various forms of Aβ, including monomer, oligomer and fibril, have been shown to coordinate with redox active transition metals, such as Cu^2+^ and Fe^3+^, which induce the formation of ROS [17], [18], [20], [21]. Although the interaction and coordination configuration of Aβ/Cu^2+^ complexes has been extensively studied [16]–[18], [29], [32]–[34], the effect of Aβ sequence, particularly C-terminal residues, on Cu^2+^ binding affinity, structural property, aggregative ability and ROS formation still remains to be elucidated.

In the present study, results demonstrate that the C-terminal residues of Aβ have significant effect on Cu^2+^ binding affinity, structure, aggregation ability and inhibitory ability of ROS. For Cu^2+^ binding affinity, the C-terminal residues of Aβ, particularly residues 25–29 and 36–40, have a strong effect on Cu^2+^ binding affinity as evidenced by the fact that the Cu^2+^ binding constants for Aβ1-40 and Aβ1-29 are higher than those for other C-terminus-truncated Aβ peptides. Even though the Cu^2+^ binding affinity is dependent on C-terminal residues of Aβ, the coordination configurations of Aβ/Cu^2+^ are not significantly altered by the interaction C-terminal residues with Cu^2+^ as the hyperfine patterns and parameters obtained from EPR spectroscopy are similar for these different Aβ peptides. The coordination configuration of Aβ/Cu^2+^ still adopt the a 3N1O mode, and His6, His13 and His14 residues are the main amino acid residues to interact with Cu^2+^ even for C-terminus-truncated peptides [32], [33]. For the Cu-binding mode, our present study shows that the Cu^2+^ binding site for all Aβ peptides is able to locate two Cu^2+^ ions. These two Cu^2+^ ions bound to Aβ is dependent on the C-terminal residues. The binding constant for the first Cu^2+^ ion is higher than that for the second Cu^2+^ ion. This is consistent with a previous study [29]. The fold difference between the first Cu^2+^-binding constant and the second Cu^2+^-binding constant is also dependent on Aβ C-terminal residues, ranged from 160 folds for Aβ1-40 to 10 folds for Aβ1-16, respectively. However, the binding constants obtained in this study are somehow lower than the previous report [29]. The possible reason may be two folds; the first reason may be due that the concentration of Aβ peptides used is lower than the previous study, and the second reason may be caused by the different method applied.

It is interesting to note that the trend of *K_a1_* and *K_a2_* is generally opposite to each other. The *K_a1_* values are higher for Aβ peptides with residues 25–29 and 36–40, whereas the *K_a2_* values for Aβ peptides with residues 25–29 and 36–40 are generally lower compared to other C-terminus-truncated peptides. This indicates that the residues 25–29 and 36–40 possibly increase the binding affinity of the first Cu^2+^ ion and decrease the binding of the second Cu^2+^ ion. This result may provide an explanation for the previous observation that the second Cu site is only observed in the shorter truncated Aβ peptides such as Aβ1-16 [35], since the C-terminal residues, particularly residues 36–40, may impede the binding of the second Cu^2+^. However, the second Cu^2+^ binding constant is rather small compared to the binding constant of the first Cu^2+^, thereby the role of second Cu^2+^ ion also has little effect on the function of Aβ such as coordination geometry. The effect of Cu-binding on Aβ function is mainly attributed from the binding of the first Cu^2+^ ion.

Besides the effect of C-terminal residues on Cu^2+^ binding affinity, the C-terminal residues also show to have impact on the structural property of Aβ in the presence of Cu^2+^. From the result of secondary structural analysis, Aβ1-40 has the highest β-sheet propensity, indicating that residues 36–40 may play a key role on structural conversion of Aβ from random coil into β-sheet driven by Cu^2+^. Previously, residues 17–21 and 30–35 have been shown to be the key regions on the conformation stability for Aβ in the absence of Cu^2+^ [14], [15]. Our present results indicate that, in the presence of Cu^2+^, residues 36–40 may be more important than residues 17–21 and 30–35 for the conformational conversion of Aβ driven by Cu^2+^. Recently, a solid-state NMR study showed that the hydrophobic core regions of residues 18–25 and 30–36 of fibril Aβ/Cu^2+^ complex have little structural change [34]. Our present result is consistent with their study.

A similar effect of C-terminal residues on Aβ aggregation was obtained in the presence of Cu^2+^, since the aggregation ability of Aβ is highly associated with the ability of structural conversion into β-sheet [14], [15]. Previous studies showed that, in the absence of Cu^2+^, residues 17–21 and 30–35 are the most important regions for aggregation and neurotoxicity of Aβ [14], [15]. The present results show that the structural feature responsible for the aggregation in the presence of Cu^2+^ is very different from that in the absence of Cu^2+^. In the presence of Cu^2+^, the residues 36–40, instead of residues 17–21 and 30–35, are the key amino acid residues responsible for the aggregation, as Aβ1-40 has the fastest aggregation rate.

The role of Aβ on the ROS production in the presence of Cu is still under debate. Both inhibition and production of ROS by Aβ/Cu complex have been proposed [21], [22], [27], [28]. However, our results show that Aβ inhibits the ROS production in the presence of Cu. Recently, Fang and his colleagues have reported that H_2_O_2_ production is highly dependent on the state of Aβ, which monomeric Aβ tends to inhibit H_2_O_2_ production in the presence of Cu, whereas Aβ oligomer and fibril in the presence of Cu can induce H_2_O_2_ production [27]. According to their finding, our result may indicate that Aβ used in the present study may exist at monomeric state.

For the inhibition of ROS production, similar sequence-effect was also observed for the inhibitory ability of ROS formation. Results show that the inhibitory ability is also well correlated with the C-terminal residues of Aβ. Aβ1-40 with the C-terminal residues 36–40 is the only peptide which can completely inhibit the H_2_O_2_ formation, whereas the other C-terminus-truncated peptides, lacking the residues 36–40, can only inhibit the level of H_2_O_2_ to a less degree. Furthermore, the inhibitory ability is also dependent on Cu^2+^ binding affinity, as the binding affinity is correlated with the length of C-terminal residues. Therefore, the stronger binding constant, the higher inhibitory ability of ROS formation is.

Previous studies showed that Aβ peptides form non-amyloidogenic aggregates in the presence of Cu^2+^ and amyloidogenic fibril in the absence of Cu^2+^ [36]–[38]. Our present study also examined the morphologies for these Aβ peptides in the presence of Cu^2+^. Results show a similar observation which all Aβ peptides with Cu^2+^ form non-amyloidogenic aggregates. After Cu^2+^ ions stripped off by EDTA, only Aβ1-40, Aβ1-35 and Aβ1-29 can form a typical amyloidogenic fibril, but both Aβ1-24 and Aβ1-16 do not form any fibril. It is of interest to note that the morphologies of amyloidogenic fibril formed by Aβ1-40 and Aβ1-29 and are slightly different from the morphology of Aβ1-35 fibril. Both Aβ1-40 and Aβ1-29 form a short, rod and non-network-like fibril, whereas morphology of Aβ1-36 fibril is a typical thin and rod- and network-like fibril. The cause of the different morphologies between these peptides is unclear, but it is correlated with the sequence of Aβ and Cu^2+^ binding affinity, since the Cu^2+^ binding affinity of Aβ1-35 is weaker than those of Aβ1-40 and Aβ1-29.

In conclusion, our present results demonstrate i that the C-terminal residues of Aβ have a significant effect on Cu^2+^ binding affinity, structure, aggregation ability and inhibitory ability of ROS formation. Among the C-terminal residues, residues 36–40 play the most important key role on these properties. The involvement of C-terminal residues 36–40, instead of residues 17–21 and 30–35, on Cu^2+^ binding affinity, β-sheet conversion, aggregation ability and inhibitory ability may provide a possible explanation for the different behavior of Aβ in the presence of Cu^2+^.
